# A Novel Stress-Associated Protein ‘AtSAP10’ from *Arabidopsis thaliana* Confers Tolerance to Nickel, Manganese, Zinc, and High Temperature Stress

**DOI:** 10.1371/journal.pone.0020921

**Published:** 2011-06-09

**Authors:** Anirudha R. Dixit, Om Parkash Dhankher

**Affiliations:** Department of Plant, Soil, and Insect Sciences, and Plant Biology Graduate Program, University of Massachusetts, Amherst, Massachusetts, United States of America; Iwate University, Japan

## Abstract

We describe here the functional characterization of a novel AtSAP10, a member of the Stress Associated Protein (SAP) gene family, from *Arabidopsis thaliana* ecotype Columbia. AtSAP10 contains an A20 and AN1 zinc-finger domain at the N- and C-terminal, respectively. *Arabidopsis SAP10* showed differential regulation by various abiotic stresses such as heavy metals and metalloids (Ni, Cd, Mn, Zn, and As), high and low temperatures, cold, and ABA. Overexpression of AtSAP10 in *Arabidopsis* conferred strong tolerance to heavy metals such as Ni, Mn, and Zn and to high temperature stress. AtSAP10 transgenic plants under these stress conditions grew green and healthy, attained several-fold more biomass, and had longer roots as compared to wild type plants. Further, while these transgenic plants accumulated significantly greater amounts of Ni and Mn in both shoots and root tissues, there was no significant difference in the accumulation of Zn. *AtSAP10* promoter-GUS fusion studies revealed a root and floral organ-specific expression of AtSAP10. Overexpression of AtSAP10-GFP fusion protein showed the localization in both nucleus and cytoplasm. Taken together, these results showed that AtSAP10 is a potentially useful candidate gene for engineering tolerance to heavy metals and to abiotic stress in cultivated plants.

## Introduction

Plants are constantly exposed to unfavorable environmental conditions such as drought, high salinity, extreme temperatures, and heavy metals. These stresses can induce various biochemical and physiological changes that result in plant growth inhibition and productivity. It is estimated that abiotic stresses can cause a 30-50% loss in crop productivity worldwide [1]. To survive under stress conditions, plants have to adjust their metabolism via regulating the expression of genes participating in stress tolerance such as transcription factors, molecular chaperones, ion channels, and transporters [2], [3]. Kanneganti and Gupta [4] suggested “tolerance to multiple stress conditions can be achieved by overexpressing transcription factor(s) that are involved in controlling multiple genes from various pathways or by overexpressing genes involved in abiotic stress signal perception and transduction.”

The Stress Associated Protein (SAP) family in recent years has emerged as an important gene family involved in multiple abiotic stress responses in plants. There are 14 and 18 reported members of the SAP family in *Arabidopsis* and rice, respectively [5]. Members of SAP family have the characteristic A20/AN1 zinc-finger domains, and are shown to play a central role in regulating the immune response [6], [7], [8], [9]. The A20 zinc-finger domain is identified as a part of TNFα-inducible protein A20 in human endothelial cells [10], and the AN1 domain is identified as a N-terminus putative zinc-finger domain in the proteins coded by the *Xenopus laevis* animal hemisphere 1 (AN1) maternal RNA [11]. In animals, two A20/AN1 zinc-finger proteins, ZNF216 and AWP1, have been extensively studied [6], [12]. ZNF216 plays a role in regulating NF-kB activation and apoptosis, and AWP1 has been suggested to function in the mammalian signal-transduction pathways. Plant SAPs contain an A20, AN1, or both A20/AN1 zinc-finger domains at the N- or C-terminal. Some SAP proteins also contain extra Cys2-His2 RING motifs at the C-terminus [13]. The A20 zinc-finger domain is characterized by multiple Cys2-Cys2 finger motifs [14], whereas, the AN1 zinc-finger domain is characterized by the presence of multiple Cys and His residues. The Cys- and His-residues in these zinc-finger domains are arranged in specific orders that also form typical metal-binding domains. Based on the phylogenetic analysis of the AN1 zinc-finger domains, Jin et al. [13] recently divided all A20/AN1 zinc-finger-containing SAP genes into two groups: Type I and Type II. Type I genes contain the traditional pattern of cysteine- and histidine-rich motifs such as CX_2_CX_9-12_CX_1-2_CX_4_CX_2_HX_5_HXC, whereas Type II SAP genes contain the expanded domain CX4CX_2_CX_9-12_CX_1-2_CX_4_CX_2_HX_5_HXC where X represents any amino acid [13], [15]. Most Type I genes lack introns and contain one intact A20 type domain and/or one AN1 type zinc-finger domain; most Type II genes have a single intron but do not contain an A20 domain.

In plants, those SAPs with A20/ANI zinc-finger domains have been suggested as playing a significant role in abiotic stress responses [3]. Most rice SAP genes are induced in response to various abiotic stresses [3], [4], [5]. So far three Type I SAP rice genes containing one A20 and one AN1 domain (*OsiSAP1*, *OsiSAP8*, and *ZFP177*: identical to *OsiSAP9*) have been cloned and overexpressed in tobacco and rice [3], [4], [16]. It has been shown that both *OsiSAP1* and *OsiSAP8* genes are induced in response to multiple environmental stresses such as cold, drought, heavy metals, wounding, and submergence. Overexpression of OsiSAP1 in tobacco causes an increased stress tolerance to salt, cold, and drought [3]; similarly, the overexpression of OsiSAP8 in rice provides strong tolerance to drought, salt, and cold [4]. Also, sub-cellular localization of OsiSAP8 GFP-fusion protein indicates that OsiSAP8 is a cytoplasmic protein. Overexpression of ZFP177, another rice zinc-finger A20/AN1 gene, in tobacco plants resulted in an increased tolerance to both high and low temperature and H_2_O_2_ stresses but on the other hand caused an oversensitivity to dehydration and salt stresses [16]. Similarly, overexpression of AlSAP, a stress-associated protein from a halophyte grass *Aeluropus littoralis*, in tobacco provides an increased tolerance to salt, drought, cold, and heat stress [15]. *AtSAP12*, another member of the SAP family in *Arabidopsis*, has shown a strong upregulation of its transcript levels as soon as 6 hours of cold and salt treatment [17]. The underlying molecular and biochemical mechanisms by which these *SAP* genes confer strong tolerance to various abiotic stresses are not known. Kanneganti and Gupta [4] have shown that the A20 and AN1 zinc-finger domains of OsiSAP8 interact with each other and suggest that *OsiSAP1* and *OsiSAP8* gene products might act early in the signal transduction pathways of stress responses and may use their zinc-finger domains for protein-protein interactions. Recently, AtSAP5, a member of the *Arabidopsis* SAP family, has been shown to act as an E3 ubiquitin ligase through its AN1 domain and provides tolerance to dehydration stress [18].

Apart from the aforementioned studies, very little is known about members of the SAP family in plants. We describe here the functional characterization of AtSAP10, a member of the SAP gene family in *A. thaliana*, which contains an A20 and AN1 zinc-finger domain at the N- and C-terminal, respectively. Overexpression of AtSAP10 in *Arabidopsis* provided strong tolerance to several toxic metals and to high temperature stress.

## Materials and Methods

### Plant materials and growth conditions

Seeds of wild type and transgenic *A. thaliana* ecotype Columbia were sterilized in 30% (v/v) bleach for 30 minutes, rinsed five times with sterile deionized water, and inoculated onto plates containing half-strength Murashige and Skoog (MS) medium with vitamins [19] (Phytotech Laboratories, KS, USA), 0.8% w/v Phytoblend agar (Caisson Laboratories, UT, USA), and 1% w/v sucrose. Seeds were stratified at 4°C for 24 hours prior to transfer to a controlled-environment cabinet (cycling 16 hours light and 8 hours dark at 22°C and 18°C, respectively) and incubated vertically after germination.

### Amplification and cloning of *AtSAP10* gene

The *AtSAP10* cDNA sequence (accession NM_118670) was PCR amplified from *Arabidopsis* flower cDNA library using the forward primer 5′-TACGTCGGATCCAGG AGGTAGACCATGGTGAACGAAACAGAAGCAT-3′ and reverse primer 5′-TAGCTGCTCGAGAAGCTTCTAAAACCTCTGCAACTTGTCA-3′. The PCR conditions used for *AtSAP10* amplification were: 2 minutes at 94°C, followed by 30 cycles of 45 seconds at 94°C, 1 minute at 55°C, 45 seconds at 72°C, and finally extending for 10 minutes at 72°C. The resulting PCR product was introduced as *Nco*I/*Xho*I fragment under the control of an *Actin2* gene promoter and terminator expression cassette (*ACT2pt)* [20] to make the construct *ACT2pt/AtSAP10* and sequences were confirmed. The sequenced construct was sub-cloned as a *Kpn*I*/Sac*I fragment into the plant binary vector pBIN19 making *pBIN19/ACT2pt/AtSAP10* (Figure S1-A). The *AtSAP10* promoter-GUS fusion construct was generated by using pBI101vector (Clontech). A 1000 bp fragment of *AtSAP10* containing putative promoter region was amplified from genomic DNA by PCR using the forward primer 5′-TAGCTGAAGCTTTCGTTACATCATGGTTTATAACG-3′ and reverse primer 5′-TAGCTG*TCTAGA*CTTCTTTCTTCTACTTCTTGCGA-3′ and cloned into *pBI101* using *Hind*III*/Xba*I sites (Figure S1-B).

For sub-cellular localization, a *pBIN19/ACT2pt/AtSAP10-eGFP* construct was made by amplifying the coding region of *AtSAP10* without the stop codon from an *Arabidopsis* flower cDNA using two specific oligonucleotide primers 5′-TACGTCGGATCCAGGAGGTAGACCATGGTGAACGAAACAGAAGCAT-3′ and 5′-TAGCTGGTCGACGCGGCCGCAAACCTCTGCAACTTGTCA-3′. The *Not*I restriction site placed before *Sal*I in the reverse primer allowed the cloning of the coding DNA in frame to the *eGFP* gene in the binary vector *pBIN19*, which contained the *ACT2p/eGFP/ACT2* terminator cassette (Figure S1-C). For control, wild type *Arabidopsis* mesophyll cell protoplasts were transformed with construct ACT2pt-eGFP expressing the *eGFP* gene alone under the *ACT2pt* expression cassette.

### Plant abiotic stress treatments to study *AtSAP10* gene regulation

For arsenite (AsIII), arsenate (AsV), zinc (Zn), and cadmium (Cd) stress treatments, wild type *Arabidopsis* seeds were grown in half-strength liquid MS medium in 250 ml flasks under control conditions (16 hours light and 8 hours dark at 22°C and 18°C, respectively) with constant swirling. After 12 days, plants were exposed to toxic metals by adding sodium arsenite at 25 µM, sodium arsenate at 150 µM, cadmium chloride at 75 µM, and zinc sulfate at 500 µM. Tissue was collected after 0, 12, and 24 hours. For nickel (Ni), manganese (Mn), and ABA treatments, seeds of wild type plants were germinated on a nylon mesh placed on half-strength MS agar plates. The 12 days old seedlings along with the supporting mesh were transferred on a 2 cm long piece of 50 ml Nalgene plastic tube support placed in the magenta boxes containing half-strength MS liquid medium and allowed to acclimatize for additional period of seven days. At the end of acclimatization period, plants were exposed to Ni, Mn, and ABA by adding 90 µM nickel chloride, 1 mM manganese chloride, and 1.5 µM ABA, respectively. Shoot and root tissues were harvested separately after 0, 6, 12, and 24 hours.

For heat stress treatment, magenta boxes containing wild type plants were kept in an incubator at 38°C for 0.5, 1, 3, and 6 hours. For cold treatment, magenta boxes with wild type plants were transferred to a growth chamber maintained at 2°C for 0.5, 1, 3, and 6 hours. Drought stress was implicated by removing plants from magenta boxes and placing them on a dry paper towel to remove any excess adhered nutrient solution. Plants were air dried by transferring into dry magenta boxes for the desired time points. Plants were exposed to salt stress by replacing normal half-strength MS medium with medium supplemented with 150 mM sodium chloride for desired time points. All samples were harvested, washed with deionized water, and stored at -80°C till further use.

### RNA isolation and RT-PCR analysis

Total RNA was extracted from hydroponically grown *Arabidopsis* plants using the RNAeasy Plant mini kit (Qiagen) according to the manufacturer's protocol. A 2 to 5 µg of total RNA was used for reverse transcription using the ThermoScript™ RT-PCR System (Invitrogen) for first-strand cDNA synthesis. The cDNA was 10-fold diluted, and PCR was performed using either Platinum® Taq DNA Polymerase (Invitrogen) or TaKaRa Ex Taq™ (Takara Bio) with gene-specific primers and an *ACT2* gene as internal control for equal cDNA loading. PCR conditions used were: 2 minutes at 94°C followed by 45 seconds at 94°C, 1 minute at 55°C, 45 seconds at 72°C, and finally extending for 10 minutes at 72°C. PCR was optimized at 30 cycles for *AtSAP10* and at 26 cycles for the internal control *ACT2* gene. All RT-PCR experiments were repeated at least three times to confirm the results.

### Plant transformation

The binary plasmids were transferred into the *Agrobacterium tumefaciens* strain C58 [21] using the heat shock method. *Arabidopsis* plants were transformed by vacuum infiltration as described previously [22]. Transgenic plants were selected on solidified half-strength MS medium containing 30 mg^−L^ kanamycin sulphate and the resulting T_1_ plants were grown to maturity. T_2_ seeds were again germinated on media with kanamycin and the independent transgenic lines showing 3∶1 (kanamycin resistant: sensitive) segregation ratio were grown to obtain the T_3_ homozygous lines which were used for further analysis.

### Plant growth assays for heavy metals tolerance/sensitivity

For heavy metal tolerance/sensitivity analysis, seeds of the wild type and transgenic plants were germinated and grown on vertically placed half-strength MS agar plates (with 1% sucrose) in the absence or presence of heavy metals for three weeks with a 16 hours light/8 hours dark cycle at 22°C/18°C day/night temperature. To reduce variations, wild type and AtSAP10 plants were grown side by side on the same plate and their growth was compared. Plants were collected, weighed and their root lengths were measured.

### Heavy metals accumulation analysis in plant tissues

For heavy metal accumulation analysis, seeds of wild type and transgenic plants were germinated on a nylon mesh placed on half-strength MS agar plates (with 1% sucrose). The 12 days old seedlings along with the supporting mesh were transferred on a 2 cm long piece of 50 ml Nalgene plastic tube support placed in the magenta boxes containing half-strength MS liquid medium and allowed to acclimatize for additional period of seven days. At the end of acclimatization period, the half-strength MS liquid medium was replaced with a new liquid medium containing the appropriate amounts of metals and were continued to grow for another four days. At the end of 4^th^ day, plants were removed from the magenta boxes, harvested root and shoot separately, washed three to four times with Milli-Q water and dried in the Kimwipes paper folds at 70°C for 48 hours.

Dried plant samples were crushed to fine powder, weighed, and then digested in the concentrated nitric acid (10 mg^−ml^) with constant shaking for 48 hours. Hydrogen peroxide (30%) was added at the end of 48 hours of acid digestion to promote oxidation of organic matter and achieve complete digestion. Samples were then centrifuged at 3000 rpm for 10 minute and the clear supernatant was diluted 10-fold with deionized water. Samples were analyzed by Elan DRCe inductively coupled plasma-mass spectrometry (ICP-MS).

### Heat stress treatments

Growth assay for tolerance to high temperature was performed using the slightly modified method of Larkindale [23]. High temperature stress tests were performed by growing wild type and transgenic plants side by side on half-strength MS agar plates (with 1% sucrose) with a 16 hours light/8 hours dark cycle at 22°C/18°C day/night temperatures for 12 days. The seedlings were initially exposed to 38°C for 90 minutes and then left at room temperature (22°C) for 2 hours before finally being exposed to 45°C for 1 hour. All heat treatments were performed in the dark. Plants were allowed to recover in a growth chamber for 6 days with a 16 hours light/8 hours dark cycle at 22°C/18°C. At the end of the 6^th^ day, plants were again heat shocked at 45°C for 3 hours and then placed in a growth chamber for a 5-day recovery period before scoring.

### Histochemical GUS assays

*Arabidopsis* tissue samples were fixed in ice-cold 90% (v/v) acetone and incubated at room temperature for 20 minutes. After the acetone was removed, tissues were stained overnight at 37°C in GUS staining solution containing 10 mM EDTA, 1 mM K_3_Fe(CN_6_), 0.1% Triton X-100, and 2 mM X-Gluc (Gold Biotech Technology; dissolved in *N*,*N*-dimethylformamide) in 100 mM sodium phosphate (pH 7.0) buffer [24]. After staining, samples were cleared with several changes of 70% ethanol. GUS-stained tissues were photographed using the Olympus SZ60 microscope fitted with a Micropublisher 5.0 RTV CCD camera (QImagaing).

### Promoter inducibility analysis by Q-PCR

To access the inducibility of AtSAP10 promoter under abiotic stress conditions, AtSAP10p-GUS transgenic plants were hydroponically grown for three weeks and were exposed to 38°C for different time intervals. RNA extraction and cDNA synthesis was performed as described in “RNA isolation and RT-PCR analysis” section. Quantitative real time PCR and calculation of the relative expression level of *β-glucuronidase* gene was performed following the instruction for Mastercycler® ep realplex (Eppendorf AG, Hamburg, Germany) with ABsolute Blue QPCR SYBR Green Mix (Thermo Fisher Scientific, Surrey, UK). PrimerQuest (Intergrated DNA Technologies) was used to design QPCR primers. *Arabidopsis EF1α* gene was used for normalization in each experiment. Relative transcript abundance was calculated using 2^−ΔΔC^_T_ method following Livak and Schmittgen [25].

### Sub-cellular localization of AtSAP10 using AtSAP10-eGFP construct

For GFP fluorescence observation, protoplasts were prepared from the leaves of one-week-old wild type and AtSAP10-eGFP plants by cutting leaves into 0.5-1 mm segments. Leaf pieces were digested in K3 medium [26] containing 1% (w/v) cellulase and 0.25% (w/v) macerozyme (Phytotech Laboratories, KS, USA) at 37°C for 12 hour. At the end of the digestion period, GFP fluorescence was determined by FITC-filtered visual inspection under a fluorescent microscope (Nikon E600 fitted with Spot RT CCD, Diagnostic Instruments). For confocal imaging, roots of 10 days old wild type and AtSAP10 plants were rinsed in Milli-Q water and immediately imaged with a Zeiss 510 laser scanning confocal microscope equipped with a Meta detector (Zeiss). GFP was excited at 488 nm of an argon laser, and the emission was collected between 500 and 530 nm. Images were edited using software provided by Diagnostic Instruments (SPOT RT Advanced) for the fluorescent microscope and by Zeiss LSM Image Browser Ver. 4.2 for the Zeiss 510 laser scanning microscope and were assembled in Adobe Photoshop.

## Results

### Differential regulation of *AtSAP10* gene in response to various abiotic stresses

**Figure 1:**
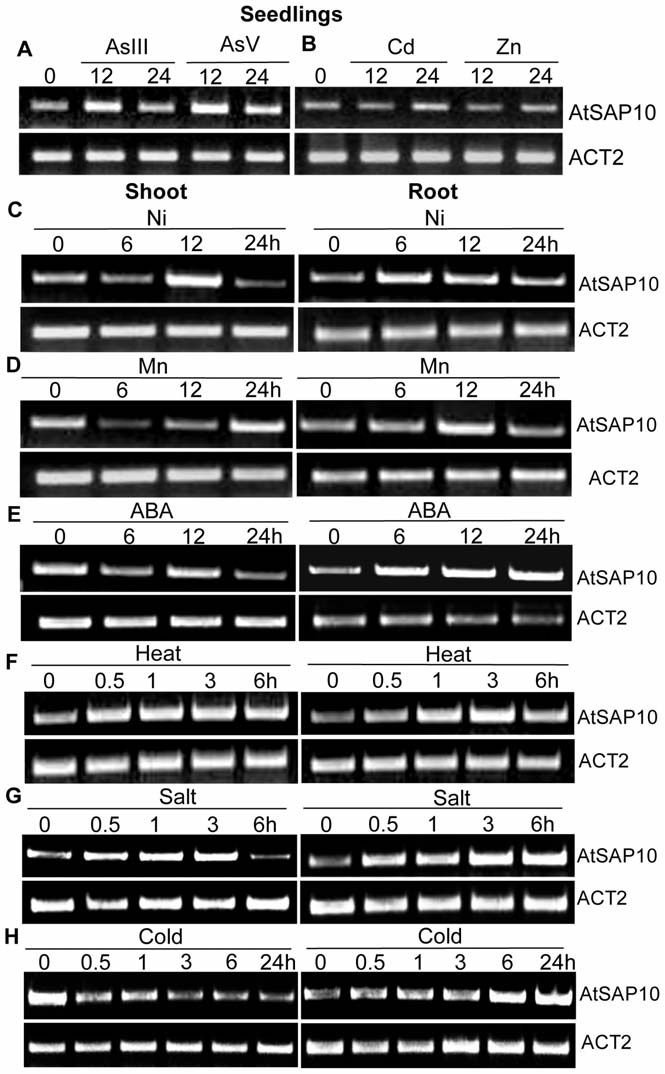
Semi-quantitative RT-PCR analysis of *AtSAP10* expression in response to various stress treatments. Expression analysis was performed with *AtSAP10* specific primers from the RNA isolated from the *Arabidopsis* seedlings subjected to As(III) and As (V) (**A**), Cd and Zn (**B**), Ni (**C**), Mn (**D**), ABA (**E**), Heat (**F**), Salt (**G**), and Cold (**H**) treatments**.** All upper panels represent *AtSAP10* and lower panel represent *ACT2* used as internal loading control. Numbers on each upper panel represents time intervals in hours for which the stress treatments were given. The presented results are the representative of at least three independent experiments.

An *in silico* analysis of 1 kb genomic sequence upstream to the transcriptional start site of *AtSAP10* using PLACE database [27] predicted the presence of several *cis*-acting regulatory elements involved in the stress responsive gene expression such as ABA responsive elements [28], drought response elements [29], W-box [30], and MYB elements [31]. The presence of these regulatory elements prompted us to test the differential regulation of *AtSAP10* in response to multiple abiotic stresses. A semi-quantitative RT-PCR was performed using the total RNA extracted from 3-weeks-old *Arabidopsis* plants subjected to various stress treatments at different time intervals as described in ‘Materials and Methods’. The time-dependent expression profiling revealed different patterns of transcript regulation for *AtSAP10* in response to various toxic metals and other abiotic stresses. Seedlings treated with AsIII, AsV, Cd, and Zn showed an increase in *AtSAP10* transcript levels after 12 and 24 hours (Figure 1A &B). Treatment with Ni and Mn also led to a significant increase in transcript levels (Figure 1C & D). In treatment with 1.5 µM ABA, *AtSAP10* transcript levels in shoots also showed a transient expression pattern but transcript levels in roots showed a strong increase at 6, 12, and 24 hours (Figure 1E).

On exposure to heat and salt stress, *AtSAP10* transcript levels in both shoots and roots increased in the first 30 minutes and continued to increase up to 6 hours (Figure 1F & G). The effect of salt exposure was less pronounced in shoots than in roots (Figure 1G). A different regulation pattern was observed in the case of cold stress. In shoots, *AtSAP10* transcript levels showed strong decrease at all time points studied, whereas in roots, strong induction was observed with a maximum at 24 hours of cold treatment (Figure 1H). Exposure to drought showed no noticeable effect to *AtSAP10* transcript levels in either shoots or root tissues (data not shown). All these results showed that *AtSAP10* is differentially regulated in response to multiple abiotic stresses. These *AtSAP10* expression patterns were consistent with the microarray expression data for abiotic stress from the AtGenExpress project [32] where the highest expression of *AtSAP10* was observed at 1, 6, and 24 hours in roots exposed to heat, salt, and cold stresses, respectively.

### AtSAP10 overexpressing plants are resistant to Ni, Mn, and Zn

To investigate the function of AtSAP10 in plants, we overexpressed the coding region of *AtSAP10* in *Arabidopsis* under the control of a strong constitutive *Actin2* promoter-terminator *(ACT2pt)* expression cassette [20]. Seeds of more than ten independent lines were screened for segregation analysis of kanamycin resistance and the lines showing 3∶1 (resistant: sensitive) ratio were selected and were grown further to select homozygous lines. Four independent T_3_ homozygous transgenic lines (AtSAP10–20, AtSAP10–23, AtSAP10–30, and AtSAP10–42) of *Arabidopsis* constitutively expressing AtSAP10 were selected for further analysis. Overexpression of *AtSAP10* was confirmed by a semi-quantitative RT-PCR analysis, and all four transgenic lines showed significantly enhanced levels of *AtSAP10* mRNA transcripts as compared to control wild type plants (Figure S2).

**Figure 2:**
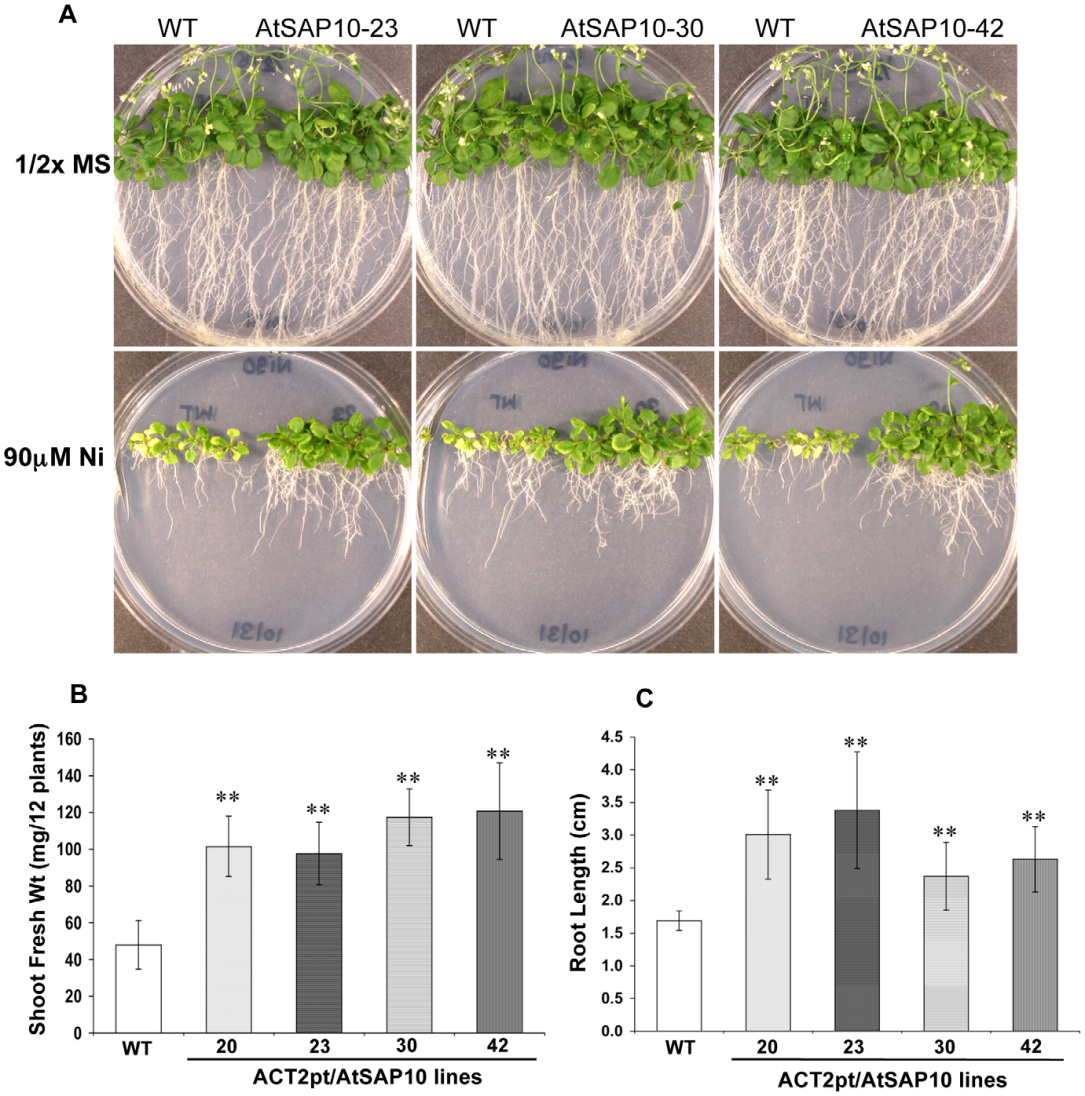
Ni resistance phenotype of *Arabidopsis* AtSAP10 overexpression lines. (**A**) Ni resistance phenotypes, (**B**) Fresh shoot weight, and (**C**) root length of three transgenic lines AtSAP10–23, AtSAP10–30, and AtSAP10–42 overexpressing AtSAP10 from *ACT2pt* expression cassette and wild type (WT) plants grown on 90 µM NiCl_2_ in half-strength MS medium for three weeks. The average and standard deviation (SD) values are represented for four replicates of 12 seedlings each for WT and all AtSAP10 lines. The asterisks represent the significant difference in biomass accumulation and root length compared with wild type (WT) plants, (*) P<0.05, (**) P<0.01.

After three weeks of growth, all four AtSAP10 transgenic lines of *Arabidopsis* showed strong tolerance to 90 µM NiCl_2_ as compared to wild type plants. The results of three representative lines are shown in Figure 2A. No phenotypic difference in growth was observed in either AtSAP10 or wild type plants grown on media without any metals. With 90 µM NiCl_2_, transgenic plants had well-developed leaves and normal roots, whereas leaves of wild-type plants were shriveled and pale and roots were stunted with minimum lateral branching. At this toxic concentration of Ni, transgenic plants attained an average 3-fold increase in shoot biomass and had significantly longer roots as compared to wild type controls (Figure 2B & C).

**Figure 3:**
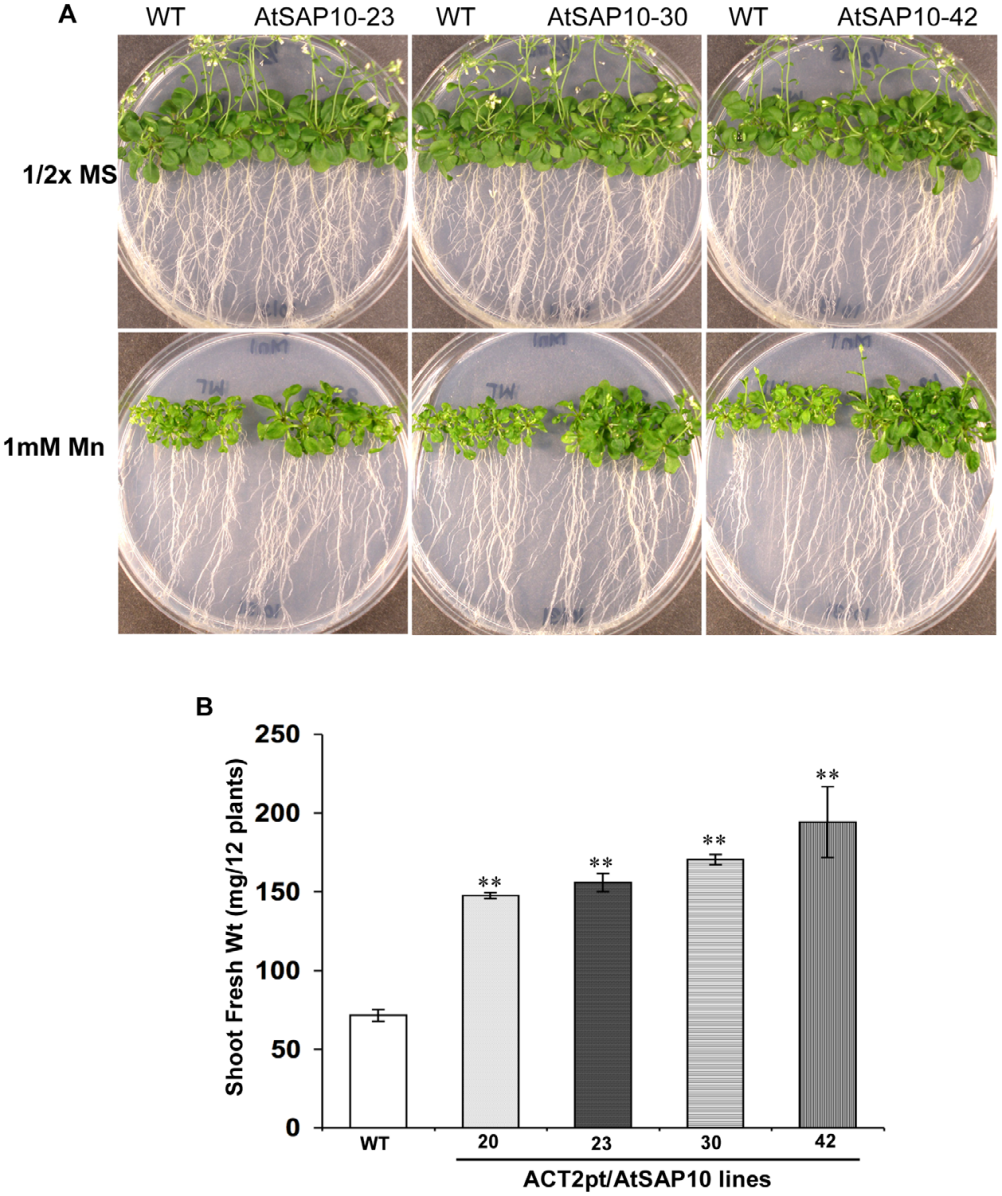
Mn resistance phenotype of *Arabidopsis* AtSAP10 overexpression lines. (**A**) Mn resistance phenotypes and (**B**) Fresh shoot weight of three transgenic lines AtSAP10–23, AtSAP10–30, and AtSAP10–42 overexpressing AtSAP10 from *ACT2pt* expression cassette and wild type (WT) plants grown on 1 mM MnCl_2_ in half-strength MS medium for three weeks. The average and standard deviation (SD) values are represented for four replicates of 12 seedlings each for WT and AtSAP10 lines. The asterisks represent the significant difference in biomass accumulation compared with wild type (WT) plants, (*) P<0.05, (**) P<0.01.

At the end of a three weeks growth period on media containing 1 mM Mn, leaves from transgenic plants were dark green and fully expanded as compared to wild-type controls whose leaves were stunted and pale (Figure 3A). AtSAP10 plants had attained a 2- to 3-fold increase in shoot biomass (Figure 3B), but no significant difference in root length was observed.

**Figure 4:**
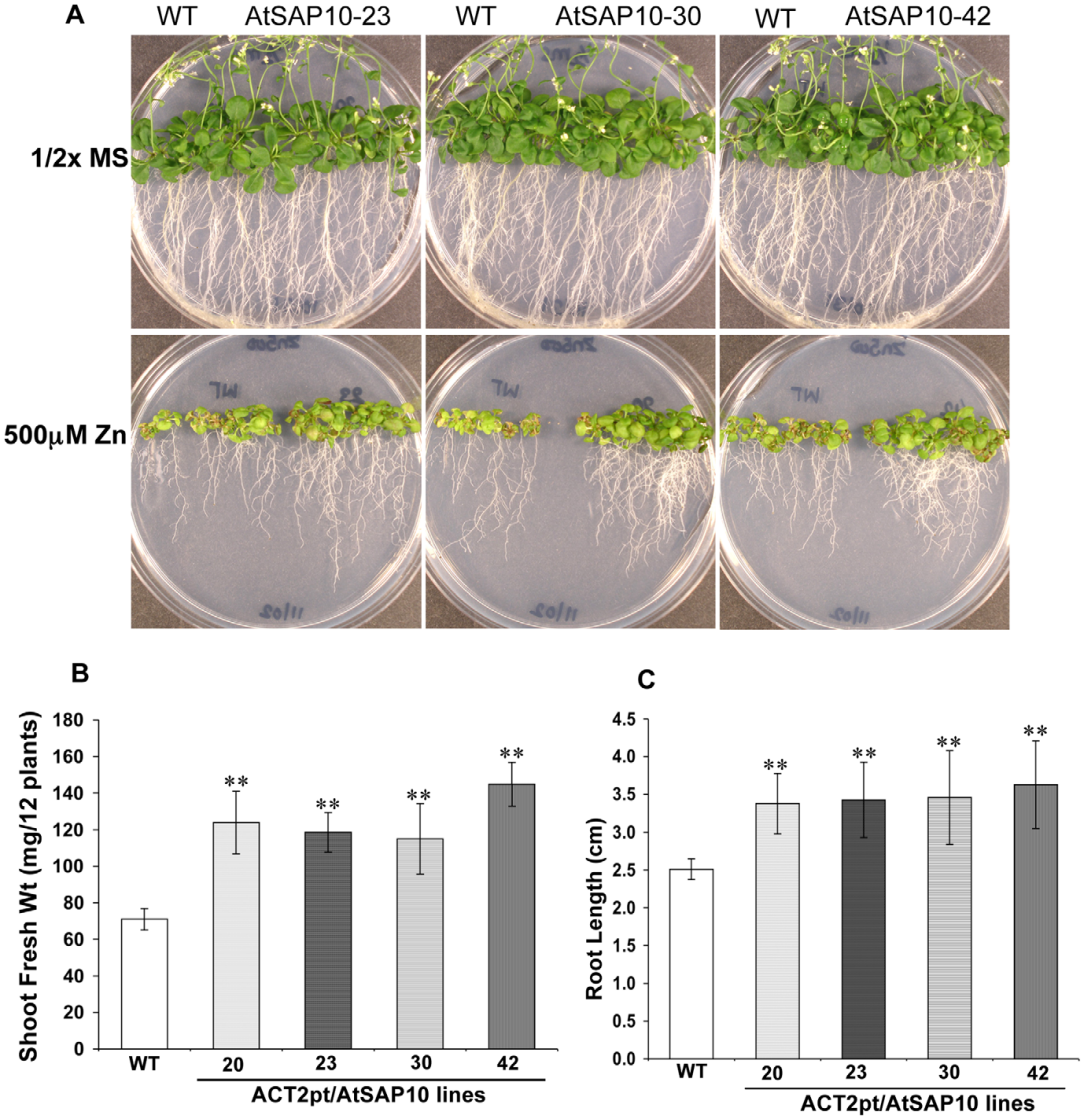
Zn resistance phenotype of *Arabidopsis* AtSAP10 overexpression lines. (**A**) Zn resistance phenotypes, (**B**) Fresh shoot weight, and **(C)** root length of three transgenic lines AtSAP10–23, AtSAP10–30, and AtSAP10–42 overexpressing AtSAP10 from *ACT2pt* expression cassette and wild type (WT) plants grown on 500 µM ZnSO_4_ in half-strength MS medium for three weeks. The average and standard deviation (SD) values are represented for four replicates of 12 seedlings each for WT and all AtSAP10 lines. The asterisks represent the significant difference in biomass accumulation and root length compared with wild type (WT) plants, (*) P<0.05, (**) P<0.01.

When exposed to 500 µM ZnSO_4_, AtSAP10 plants had well-branched roots that were longer than roots of the wild type. AtSAP10 plants had well developed green leaves compared to the wild type that had smaller leaves, pale yellow or pinkish in appearance, which eventually died at 500 µM ZnSO_4_ (Figure 4A). These plants also had significantly greater shoot biomass and longer roots than the wild type control plants (Figure 4B & C). We also analyzed the AtSAP10 overexpressing transgenic plants for their tolerance to AsV, AsIII, and Cd. Compared to the wild type controls, the AtSAP10 plants failed to show any significant difference in growth in response to As and Cd stress (data not shown).

### AtSAP10 overexpressing plants accumulate Ni and Mn

**Figure 5:**
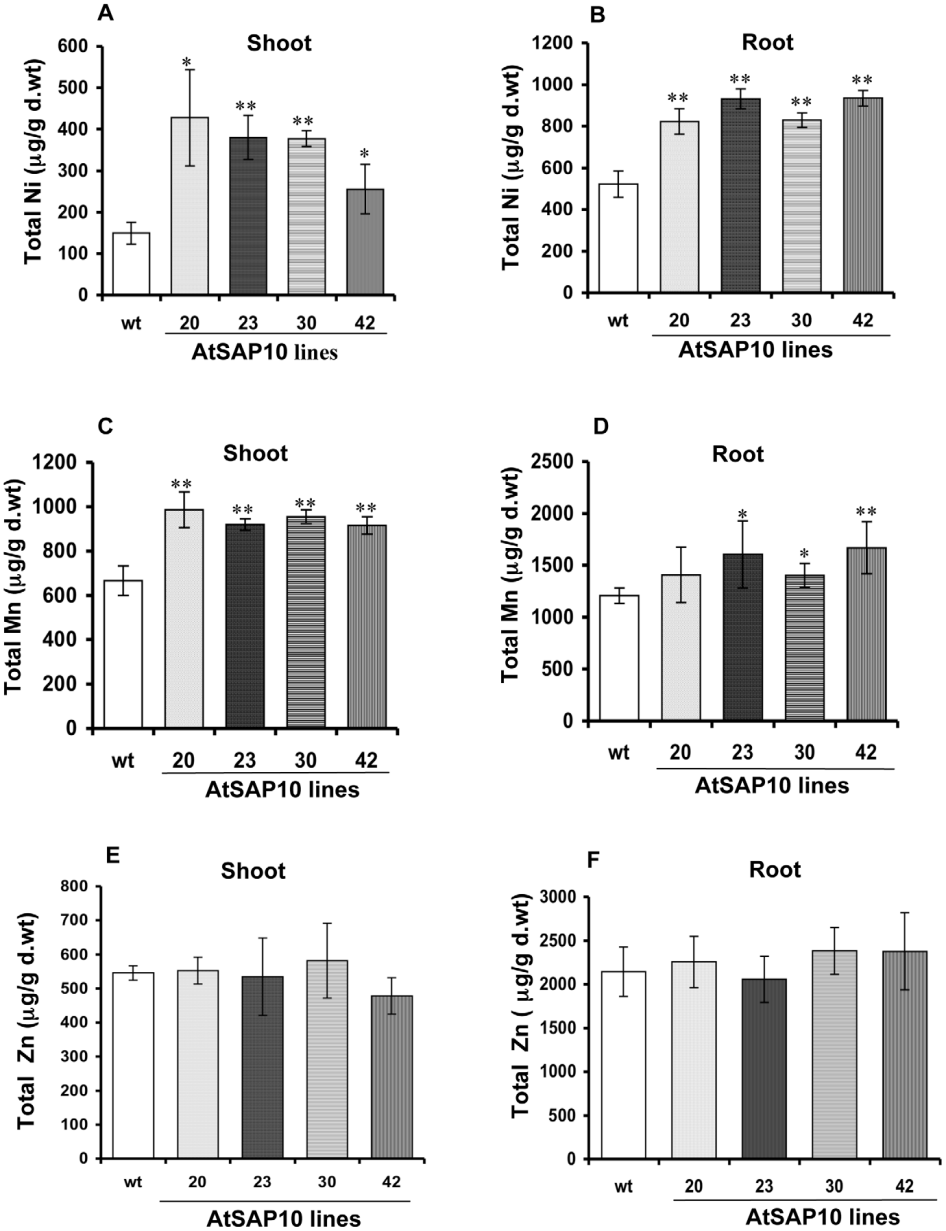
Analysis of total Ni, Mn, and Zn accumulation in *Arabidopsis* SAP10 overexpression lines. The total Ni concentration in shoots (**A**) and roots (**B**) of wild type (WT) and four overexpression lines of AtSAP10 grown on hydroponics medium containing 90 µM NiCl_2_. Total Mn accumulation in shoots (**C**) and roots (**D**) of wild type (WT) and four overexpression transgenic lines of AtSAP10 grown on hydroponics medium containing 1 mM MnCl_2_. Total Zn accumulation in shoots (**E**) and roots (**F**) of wild type (WT) and four overexpression transgenic lines of AtSAP10 grown on hydroponics medium containing 500 µM ZnSO_4_. The average and standard deviation (SD) values are shown for four replicates of 25 plants each for WT and all AtSAP10 lines. Asterisk represents the significant difference in Ni, Mn, or Zn accumulation as compared to wild type (WT), (*) <0.05, (**) <0.01.

Because AtSAP10 overexpression provided strong tolerance to heavy metals such as Ni, Mn, and Zn (as described above), we analyzed these plants for the accumulation of metals in shoot and root tissue. All four lines of the AtSAP10 transgenic and wild type *Arabidopsis* plants were grown hydroponically and elemental analysis was estimated for Ni, Mn, and Zn content in the roots and shoots. When grown on 90 µM Ni for four days, AtSAP10 overexpressing plants, had accumulated 2- to 3-fold more Ni in shoots and almost 2-fold more Ni in roots as compared to wild type plants (Figure 5A & B). When grown on 1 mM Mn, the AtSAP10 overexpressing lines had significantly greater amounts of Mn in their shoots with slightly less but still significantly greater amounts of Mn in their roots than did the wild type plants (Figure 5C & D). When analyzed for Zn, AtSAP10 plants did not show any significant uptake in either shoot or root tissue as compared to the wild type controls (Figure 5E & F).

### AtSAP10 overexpressing plants are resistant to high temperature stress

**Figure 6:**
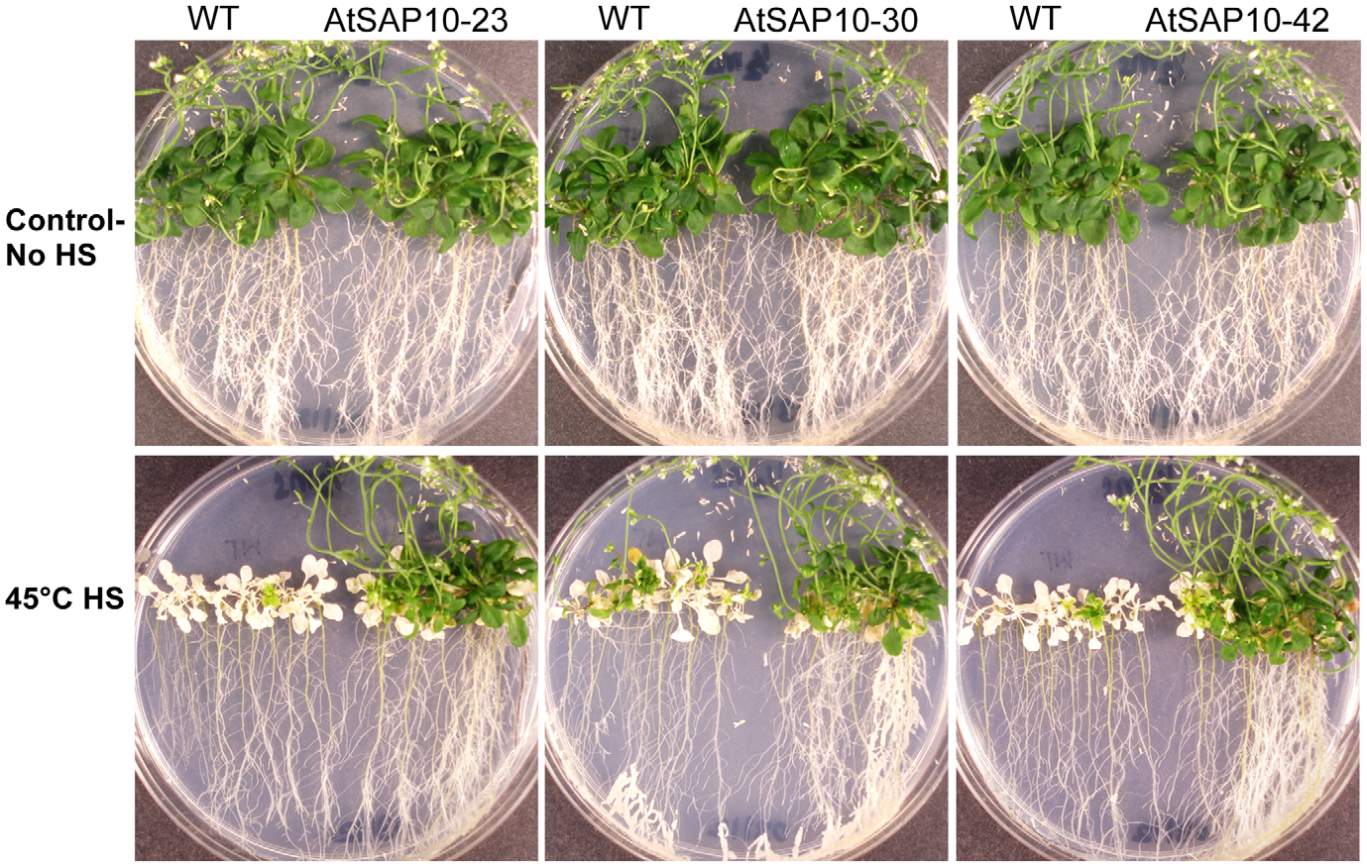
Effect of high temperature stress on wild type and AtSAP10 overexpression transgenic lines. 12-day old seedlings were heat stressed (HS) as discussed in the ‘Materials and Methods’ section. Photographs of representative plates containing wild type (WT) and AtSAP10 transgenic lines were taken after 5 days of recovery.

To determine the effect of AtSAP10 overexpression on high temperature stress tolerance, seeds of the AtSAP10 transgenic and wild type plants were grown and 12 days old seedlings were heat stressed as described above. Plants were examined at the end of the 5 days recovery period following the final heat-shock treatment. Plants that were still green and producing new leaves were scored as surviving. After 5 days of recovery, all wild type plants were dead and no new leaves had formed. In the AtSAP10 transgenic lines, however, most plants were still green and had formed new leaves, thus showing a minimum effect of heat damage (Figure 6). These results show that AtSAP10 provides strong tolerance to high temperature stress.

### Analysis of an AtSAP10 T-DNA insertion line for sensitivity/tolerance to heavy metals and heat shock stress

A semi-quantitative RT-PCR analysis of the T-DNA insertion line (SALK_036061C) with an insertion in the 5′ UTR of the *AtSAP10* gene from ABRC [33] showed no reduction in the transcripts level (Figure S3). Seeds of mutant homozygous line showed no phenotype when grown on toxic metals and under high temperature stress condition (data not shown).

### Tissue-specific expression analysis of AtSAP10

**Figure 7:**
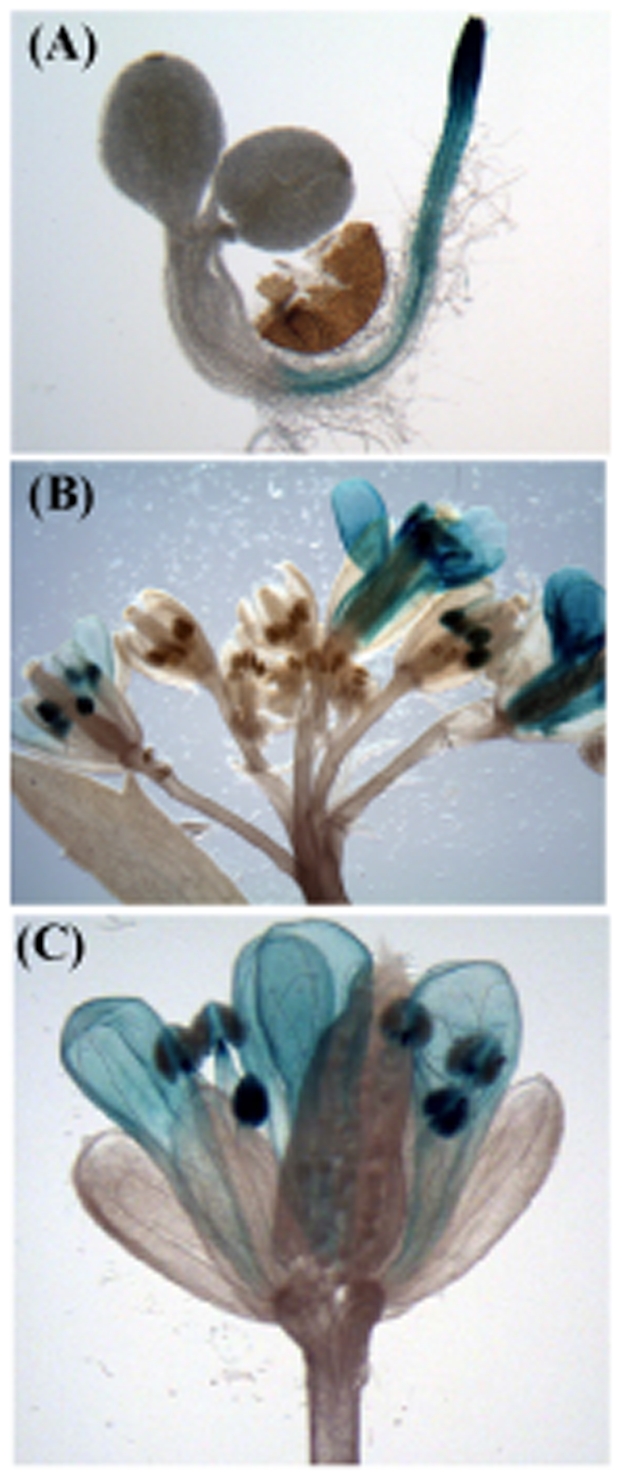
Tissue-specific expression pattern of AtSAP10 as a transcriptional fusion of the GUS reporter gene to the promoter of *AtSAP10*. Two days old seedlings of AtSAP10p-GUS (**A**); inflorescence of AtSAP10p-GUS (**B**); a flower of AtSAP10p-GUS (**C**). Scale bar, 1 mm.

To determine the tissue-specific expression of *AtSAP10* gene, we created transgenic lines carrying the GUS gene fused to the 1 kb promoter region of *AtSAP10*. Histochemical analysis showed prominent GUS staining in roots (Figure 7A) and in floral parts such as petals, stamens, and anthers (Figure 7B & C). GUS localization in the roots showed a clear demarcation between the epicotyl and hypocotyls. No GUS staining was observed in epicotyl, stems, and leaves of AtSAP10p-GUS transgenic lines at any developmental stages. A qPCR analysis of GUS transgenic plants exposed to high temperature stress showed a 2- to 3-fold increase in the levels of *β-glucuronidase* transcripts compared to untreated AtSAP10p-GUS lines (Figure S4). These expression results were in agreement with the microarray data available online (https://www.genevestigator.ethz.ch/). Developmental data from the AtGenExpress project [34], for example, also showed greatest *AtSAP10* transcript levels in the floral organs and roots.

### Sub-cellular localization of AtSAP10-GFP fusion protein

**Figure 8:**
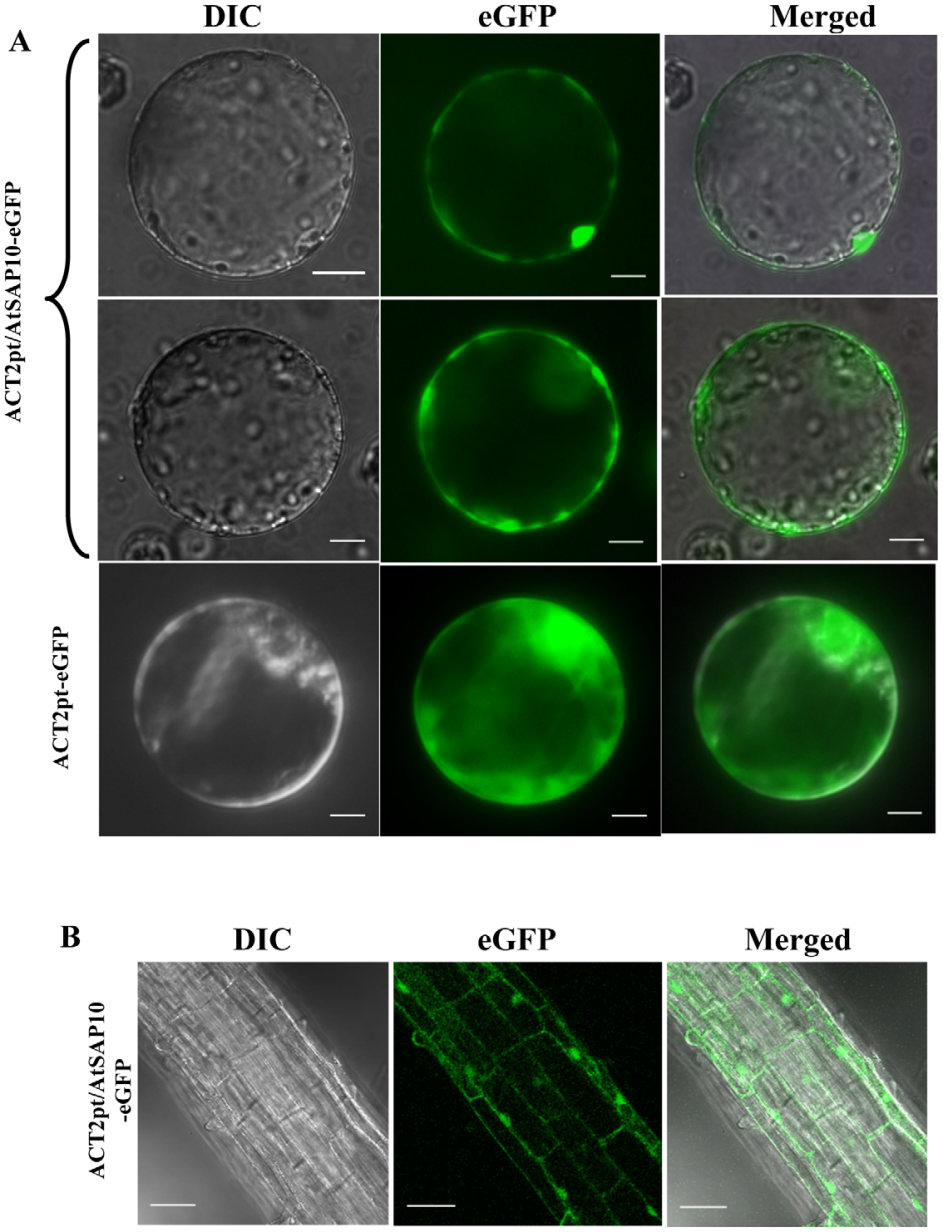
Localization of GFP-tagged AtSAP10 in *Arabidopsis*. (**A**) GFP expression in protoplasts: Upper Panel (i, ii, and iii) and middle panel (iv, v, and vi) showing the GFP fluorescence in protoplasts from plants overexpressing AtSAP10-GFP fusion protein in two different planes; lower panel showing the GFP fluorescence in protoplasts transformed with ACT2pt-eGFP as control. Scale bar, 10 µm. (**B**) Confocal laser scanning microscopy image of AtSAP10-GFP fusion protein of the root tissues: panel (i, ii, and iii) showing the GFP fluorescence in the roots of AtSAP10-GFP fusion plants. Scale bar, 20 µm.

The WoLF PSORT protein localization program [35] predicated a nuclear localization of the AtSAP10. In order to confirm sub-cellular localization, we transformed wild type *Arabidopsis* with the AtSAP10-eGFP fusion construct, *pBIN19/ACT2pt/AtSAP10-eGFP*, which constitutively expressed AtSAP10-eGFP under the control of the *ACT2pt* expression cassette. Sub-cellular localization of the chimeric protein was then analyzed using a fluorescent microscope (Figure 8A). The upper panel (i, ii, iii) showed the AtSAP10-eGFP fluorescence exclusively within the nucleus of the protoplast. When observed under a different plane, the protoplasts showed a typical cytoplasmic localization of the AtSAP10-eGFP fusion protein in the leaf protoplasts (middle panel- iv, v, and vi). *Arabidopsis* protoplasts transiently expressing ACT2pt-eGFP, used as control, showed the GFP expression throughout the protoplasts (lower panel- vii, viii, ix). To further confirm this observation, we used confocal laser scanning microscopy and examined the whole root sections. The AtSAP10-eGFP fusion protein localization was confirmed in the cytoplasm as well as in the nucleus of the roots cells (Figure 8B).

## Discussion

In the present study, we report a functional characterization of the *AtSAP10* gene. We have shown that *AtSAP10* is differentially regulated by heavy metals (AsIII, AsV, Cd, Ni, Zn, and Mn), heat, cold, salt, and ABA. Members of rice SAP family are also shown to be induced by multiple abiotic stresses, such as salt, drought, cold, desiccation, submergence, wounding, ABA, and heavy metals [4], [3], suggesting a role of *OsiSAP* genes as a key component of stress response in rice [5]. Within 30 minutes of the stress treatments to one week-old seedlings of *indica* rice caused an enhanced accumulation of transcripts for both *OsiSAP1* and *OsiSAP8 genes*. Abiotic stress treatments we studied showed that induction of *AtSAP10* started within the first 30 minutes to 1 hour, continued to increase at various time points, and then decreased within 24 hours. This kind of differential regulation of *AtSAP10* on exposure to various stressors such as heavy metals, salinity, drought, cold- and heat-shock suggests that its product might be required during early phases of stress response.

Our results further show that overexpression of AtSAP10 provides strong tolerance to toxic levels of Ni, Mn, and Zn. Although OsiSAP1 and OsiSAP8 [3], [4] have previously been shown to upregulate transcript levels in response to heavy metals, our study is the first to show that plants overexpressing a SAP gene exhibit strong tolerance to toxic levels of various heavy metals. Transgenic plants accumulated significantly higher amounts of Ni and Mn but not Zn than did the wild type controls. Metal tolerance and accumulation could perhaps be attributed to the presence of multiple Cys- and His-residues in the A20 and AN1 domains arranged in a pattern of C_x2_C_x9-12_C_x1-2_C_x4_C_x2_H_x5_H_x_C, which could bind to metals and thus provide tolerance and accumulation. A similar effect pertaining to the presence of multiple Cys- and His-residues has also been observed in case of AtHMA4 [36], which possesses a long C-terminus harboring a number of putative heavy metal-binding motifs made up of thirteen Cys pairs and eleven His residues. Expression of AtHMA4's C-terminus containing potential metal binding sites in tobacco caused an increased Cd and Zn concentrations in roots and shoots up to 4-fold as compared to wild type plants [37]. The arrangement of conserved Cys and His residues in A20 and AN1 zinc finger domains of AtSAP10 may have differential metal binding affinity to different metal cations and oxyanions, and thus provides selective tolerance to different metals. Further, this differential metal-binding affinity and metal stoichiometry may also cause differential accumulation of selective metals.

In observing the performance of AtSAP10 overexpressing plants under stress, we found that AtSAP10 conferred strong tolerance to high temperature stress. Previously, two other SAP genes, *OsSAP9* and *AlSAP*, have been reported to confer tolerance to high temperature stress [16], [15]. Both OsSAP9 and AlSAP when overexpressed in tobacco provided tolerance at 55°C for 4 hours and 55°C for 2.5 hours, respectively. According to Huang et al. [16], the high temperature stress tolerance provided by OsSAP9 is due to the presence of heat-shock element (HSE) in the promoter region. *In silico* analysis of 1 kb genomic sequence upstream to the transcriptional start site of *AtSAP10* using PLACE database predicted the presence of a CCAAT box suggested to be involved in increasing the heat-shock promoter activity by coordinating with the HSE [38], [39]. In 2008, Huang et al. [16] reported presence of HSEs in the promoter regions of six of the 12 rice A20/AN1-type zinc finger protein genes. Therefore, the possibility of more than one A20/AN1-type zinc finger protein playing roles as downstream effectors of heat-shock factors in heat stress responses cannot be ruled out [16].

A T-DNA insertion line with an insertion in the 5′ UTR of *AtSAP10* gene failed to shown any difference in the tolerance or sensitivity to toxic metals and heat-shock stress. Since the RT-PCR analysis showed no decrease in the *AtSAP10* transcript levels, we suggest that this could be due to the incomplete knockdown of *AtSAP10* gene. An RNAi approach to knockdown the *AtSAP10* transcripts is recommended to study the exact function of AtSAP10 in plants.

Tissue-specific expression analysis using the promoter-GUS fusion construct showed that the expression of AtSAP10 was predominantly localized to roots and floral organs such as stamens and petals. In roots, AtSAP10 was predominantly expressed in the region of the root tip, including root cap, meristem, and the elongation zone. Because roots provide primary perception in case of drought, salt, and heavy metal stresses, AtSAP10's root-specific expression suggests a role in stress tolerance. *In silico* analysis of *AtSAP10* gene product did not predict any potential nuclear localization signal, but the protein localization predictor, WoLF-PSORT [35] predicted a nuclear localization of AtSAP10 protein. In overexpressing AtSAP10-eGFP fusion protein in *Arabidopsis,* we found the fusion protein was localized to both nucleus and cytoplasm. Recently characterized AtSAP5 has also been shown to localize in the nucleus [18]. The cytoplasmic localization of AtSAP10 is in agreement with the localization of OsiSAP8 [4] and ZFP177/OsSAP9 [16] in rice. Although our results confirm the nuclear localization of AtSAP10 as predicted by the WoLF PSORT program, the GFP-fusion proteins of smaller sizes could have diffused to the nucleus; thus the possibility of a false localization of AtSAP10-eGFP to nucleus cannot be ruled out. Several studies have shown that relatively small GFP fusion protein, less than 50 kDa, diffuses through the nuclear pore complex [40], [41], [42].

An *in silico* analysis of the *AtSAP10* gene product by Pfam [43] database predicted a DNA binding function through the N-terminal A20 domain. Therefore, the possibility of AtSAP10 functioning as a transcription factor cannot be ruled out, which needs further investigations. As suggested by Kanneganti and Gupta [4], AtSAP10 may be functioning by using its A20/AN1 domains for protein-protein interactions. Further, a recent report showed that the *Arabidopsis* SAP5 act as an E3 ubiquitin ligase [18]. Based on the domain similarity to AtSAP5, the function of AtSAP10 as an E3 ubiquitin ligase needs to be determined in the future studies.

In conclusion, we showed that the AtSAP10 was found to be coding for a nuclear/cytoplasmic protein that might act early in the signal transduction of various stress responses. Overexpression of AtSAP10 in *Arabidopsis* led to enhanced tolerance to toxic levels of heavy metals and to high-temperature stress. Using promoter-GUS fusion methods, tissue-specific expression of AtSAP10 revealed the root and floral organ-specific expression of AtSAP10 protein. The exact mechanism by which AtSAP10 provides tolerance to multiple stresses is not known yet. However, overexpression of AtSAP10 and its homologs may prove useful for enhancing tolerance to various stresses in the food, forage, and bioenergy crops and thus enable these crops to be grown on marginal and/or nutrient-poor soils. Our future efforts will explore the identification of the possible interacting partners of AtSAP10 and their biochemical and molecular mechanisms in providing tolerance to various abiotic stresses.

## Supporting Information

Figure S1 **Diagrams of AtSAP10 constructs.** **(A)** Physical map of AtSAP10 gene cloned under the control of *ACT2* promoter-terminator expression cassette, *ACT2pt*, in binary vector *pBIN19* to make plasmid *pBIN19/Act2pt/AtSAP10* for plant transformation. **(B)** Map of GUS gene fused with the putative promoter region of *AtSAP10* in *pBI101* vector to make construct *pBI101/AtSAP10p/GUS* for GUS histochemical assays. **(C)** Map of AtSAP10 fused with eGFP, cloned under the constitutive *ACT2pt* expression cassette in *pBIN19* to make construct *pBIN19/Act2pt/AtSAP10-eGFP*.
(TIF)

Figure S2 **Transcript analysis of** ***AtSAP10*** **in wild type and overexpression lines.** Upper panel represents *AtSAP10* and lower panel represents *EF1α* as internal loading control.
(TIFF)

Figure S3 **Transcript analysis of** ***AtSAP10*** **in wild type and** ***atsap10*** **T-DNA insertion line (SALK_036061C) of** ***Arabidopsis*** **.** Upper panel represents *AtSAP10* and lower panel represents *Actin2* (*ACT2*) as internal loading control.
(TIFF)

Figure S4 **Relative expression of** ***β-glucuronidase*** **gene in AtSAP10p-GUS transgenic lines exposed to high temperature (38°C) for 0, 0. 5, 1 2, and 3 hours.** *Arabidopsis EF1α* gene was used for normalization of gene expression.
(TIFF)
