# Supplementary figures and images for: A Novel Stress-Associated Protein ‘AtSAP10’ from Arabidopsis thaliana Confers Tolerance to Nickel, Manganese, Zinc, and High Temperature Stress
Source: PLoS One. 2011 Jun 9;6(6):e20921. doi: 10.1371/journal.pone.0020921 (PMC3111467; doi:10.1371/journal.pone.0020921)

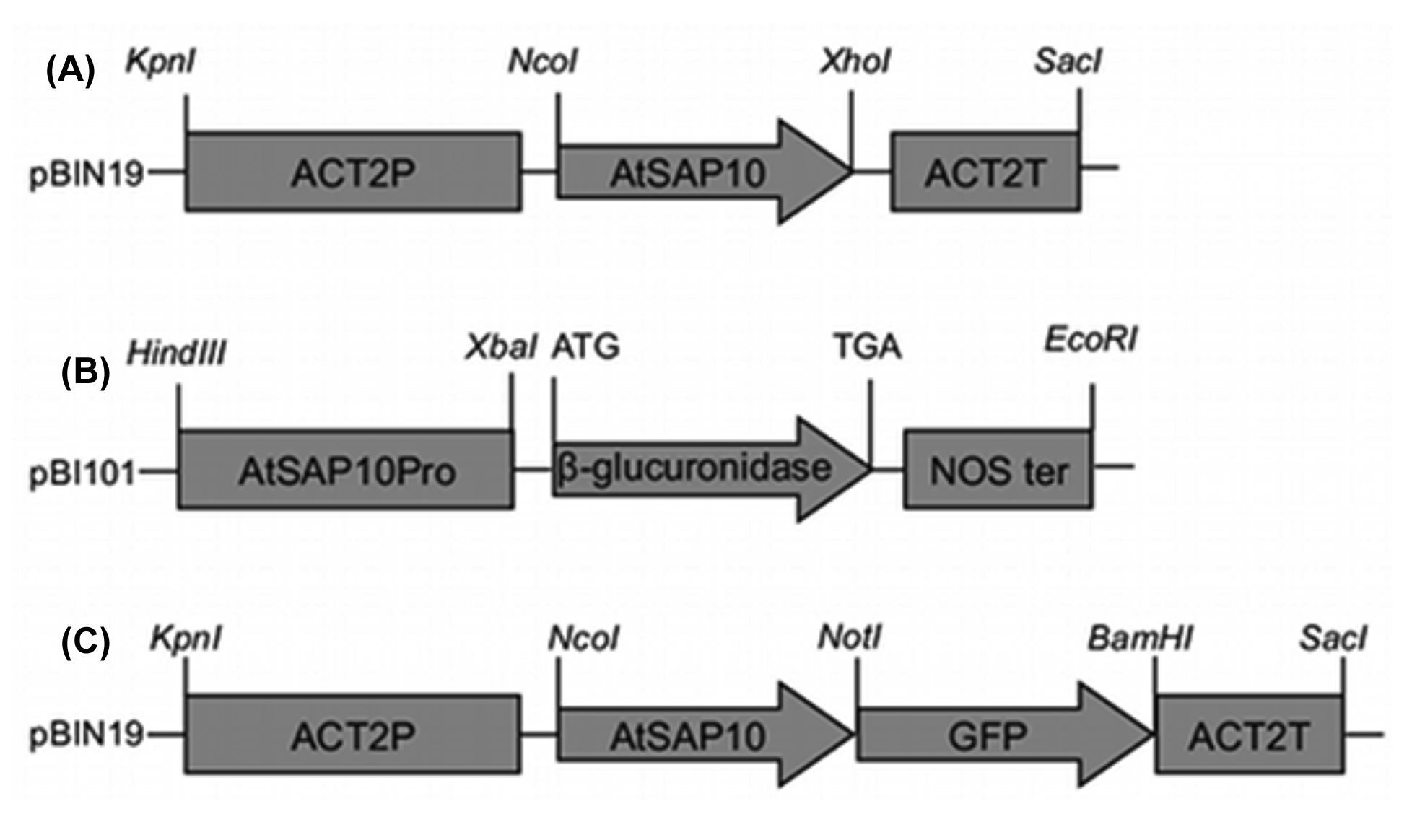

Supplement: Figure S1 — Diagrams of AtSAP10 constructs. (A) Physical map of AtSAP10 gene cloned under the control of ACT2 promoter-terminator expression cassette, ACT2pt, in binary vector pBIN19 to make plasmid pBIN19/Act2pt/AtSAP10 for plant transformation. (B) Map of GUS gene fused with the putative promoter region of AtSAP10 in pBI101 vector to make construct pBI101/AtSAP10p/GUS for GUS histochemical assays. (C) Map of AtSAP10 fused with eGFP, cloned under the constitutive ACT2pt expression cassette in pBIN19 to make construct pBIN19/Act2pt/AtSAP10-eGFP. (TIF) [file pone.0020921.s001.tif]

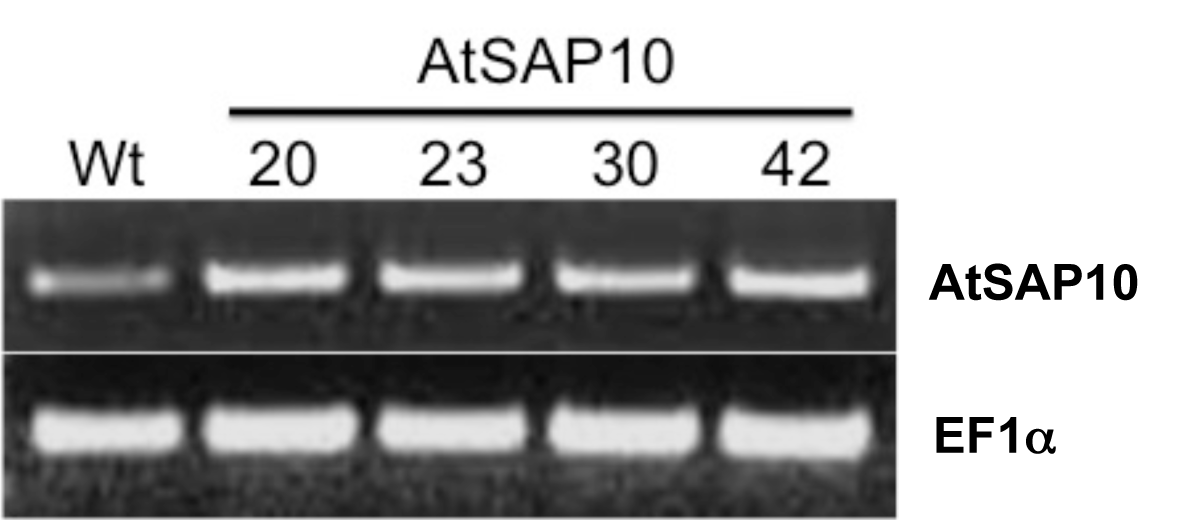

Supplement: Figure S2 — Transcript analysis of AtSAP10 in wild type and overexpression lines. Upper panel represents AtSAP10 and lower panel represents EF1α as internal loading control. (TIFF) [file pone.0020921.s002.tif]

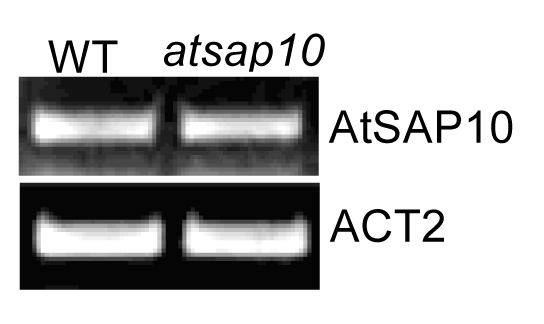

Supplement: Figure S3 — Transcript analysis of AtSAP10 in wild type and atsap10 T-DNA insertion line (SALK_036061C) of Arabidopsis . Upper panel represents AtSAP10 and lower panel represents Actin2 (ACT2) as internal loading control. (TIFF) [file pone.0020921.s003.tif]

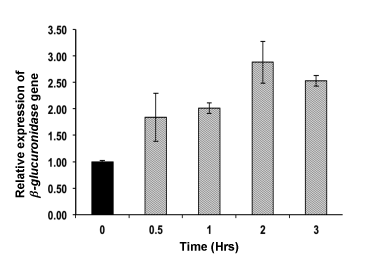

Supplement: Figure S4 — Relative expression of β-glucuronidase gene in AtSAP10p-GUS transgenic lines exposed to high temperature (38°C) for 0, 0. 5, 1 2, and 3 hours. Arabidopsis EF1α gene was used for normalization of gene expression. (TIFF) [file pone.0020921.s004.tif]
